# CP40 from *Corynebacterium pseudotuberculosis* is an endo-β-*N*-acetylglucosaminidase

**DOI:** 10.1186/s12866-016-0884-3

**Published:** 2016-11-08

**Authors:** Azadeh Shadnezhad, Andreas Naegeli, Mattias Collin

**Affiliations:** Department of Clinical Sciences, Division of Infection Medicine, Lund University, Biomedical Center B14, SE-22184 Lund, Sweden

**Keywords:** CP40, EndoS, IgG, *Corynebacterium pseudotuberculosis*, Glycosidase, Chitinase, endo-β-*N*-acetylglucosaminidase

## Abstract

**Background:**

*C. pseudotuberculosis* is an important animal pathogen that causes substantial economical loss in sheep and goat farming. Zoonotic infections in humans are rare, but when they occur they are often severe and difficult to treat. One of the most studied proteins from this bacterium, the secreted protein CP40 is being developed as a promising vaccine candidate and has been characterized as a serine protease. In this study we have investigated if CP40 is an endoglycosidase rather than a protease.

**Results:**

CP40 does not show any protease activity and contains an EndoS-like family 18 of glycoside hydrolase (chitinase) motif. It hydrolyzes biantennary glycans on both human and ovine IgGs. CP40 is not a general chitinase and cannot hydrolyze bisecting GlcNAc.

**Conclusion:**

Taken together we present solid evidence for re-annotating CP40 as an EndoS-like endoglycosidase. Redefining the activity of this enzyme will facilitate subsequent studies that could give further insight into immune evasion mechanisms underlying corynebacterial infections in animals and humans.

## Background

Glycoproteins are essential for most biological pathways in eukaryotes and play important roles in the immune system of mammals [1, 2]. Glycan chains are important for maintenance of the structure of glycoproteins and their biological function. Alterations in glycosylation pattern on glycoproteins involved in the immune system are associated with disorders such as autoimmunity and cancer [3, 4]. Immunoglobulin G (IgG) is the most abundant immunoglobulin in serum and plays a fundamental role in the adaptive immune response. The hinge and CH2 regions in IgG are involved in binding to both IgG-Fc receptors (FcγR) and the complement factor C1q (3). There is a conserved *N-*linked glycosylation site on asparagine 297 in the CH2 domain of Fc of human IgG [5]. This N*-*297 glycosylation is essential for fine tuning IgG effector functions such as binding to FcγRs and complement activation [6–8].

Glycosidases are enzymes that catalyze hydrolysis of glycosidic linkages in glycan chains. There are two kinds of glycosidases: exoglycosidases that cleave terminal carbohydrates from glycan structures, and endoglycosidases that hydrolyze linkages within the glycan chains. Numerous glycosidases from bacterial pathogens with activity on mammalian glycoproteins have been identified and characterized [9]. Some of these glycosidases are used for glycan engineering and glycan analysis of glycoproteins, or as tools for glycobiology research and drug development [10]. Endoglycosidases F_1_, F_2_ and F_3_ are important glycosidases from *Elizabethkingia meningoseptica* (formerly *Flavobacterium meningosepticum*) have been used as standard tools for glycoprotein characterization and glycoengineering. These endo-β-*N*-acetylglucosaminidases hydrolyze β-1,4-*N-*acetyl-*D*-glucosamine linkages in the chitobiose core of *N-*glycans in glycoproteins [11]. Although PNGase F is an amidase and not a glycosidase, it is often used in this context as an important tool able to release most eukaryotic N-glycans by catalyzing the hydrolysis of the amide bond between the reducing end *N*-acetylglucosamine (GlcNAc) and the asparagine residue of the glycoprotein [10, 11].

EndoS is a 108 kDa endo-β-*N*-acetylglucosaminidase belonging to family 18 of glycoside hydrolases (GH18) (CAZy, 2016; http://www.cazy.org). It is secreted by the strictly human pathogen *Streptococcus pyogenes* [12] and cleaves the β-1,4 linkage between the two GlcNAcs in the chitobiose core of the *N*-linked glycan of IgG [13]. IgG is the preferred substrate for EndoS, and this enzyme hydrolyzes the *N*-linked glycan only on native, but not denatured IgG [14]. This hydrolysis of IgG glycans most likely contributes to immune evasion by *S. pyogenes* during colonization and infection [15, 16]. In addition, based on the specificity of EndoS, purified enzyme has been used with success to remove complex *N-*linked oligosaccharide structures from the Fc region both in vitro and in vivo [16–23].

*Corynebacterium pseudotuberculosis* causes ovine caseous lymphadenitis (CLA), an infectious and contagious disease in sheep and goats. CLA is characterized by formation of abscesses in superficial lymph nodes and lesions in subcutaneous tissues. The necrotic lesions can also develop internally in spleen, kidneys, lungs, and liver. These infections can reduce meat, wool, and milk production, and are a major cause of economic loss in small ruminant farming [24]. *C. pseudotuberculosis* is mainly considered an animal pathogen, but can occasionally cause lymphadenitis in humans [25]. In 1994, Walker and colleagues identified a 40 kDa protein secreted by this bacterium and subsequently showed that vaccination of sheep with this protein provides a high level of protection against ovine caseous lymphadenitis CLA [26]. It has been shown that this 40 kDa protein, denoted CP40, is one of the predominant antigens recognized at day 7 of infection by *C. pseudotuberculosis* in sheep [26]*.* Subsequent biochemical analysis of recombinantly expressed CP40 including gelatin zymography and inhibition of enzymatic activity using protease inhibitors, have suggested that it has serine protease activity [27], but no further studies of the enzymatic activity have been conducted.

Since there are sequence similarities between CP40 and endoglycosidases such as EndoS, we hypothesized that the annotation of CP40 as a protease is incorrect, and that this major antigen of *C. pseudotuberculosis* is rather an endoglycosidase with putative activity on host glycoproteins. This hypothesis is also supported by recent a comparative genomics study on *C. ulcerans* [28], but no experimental evidence has been presented to date.

## Methods

### Bacterial isolates and growth conditions

The *C. pseudotuberculosis* type strain DSM-20689 (also ATCC 19410 and NCTC 3450) was originally recovered from infected lymphoid tissue from a sheep (DSMZ, Braunschweig, Germany). Bacteria were cultured in brain heart infusion (BHI) medium (Oxoid, Hampshire, England) supplemented with 0.5 % glucose with aeration at 37 °C for 24 h. *Escherichia coli* chemically competent strains Top10 (Invitrogen, Hämeenlinna, Finland) and BL21 (DE3) pLysE (Life Technologies, Carlsbad, CA) were propagated on luria broth (LB) agar. For selection of *E. coli* Top10, 100 μg ml^−1^ carbenicillin was added to medium and for *E. coli* BL21 in addition of 100 μg ml^−1^ carbenicillin, 34 μg ml^−1^ chloramphenicol was added. *E. coli* strains were propagated in LB overnight at 37 °C with aeration. Transformation was carried out according to manufacturer’s instructions.

### Recombinant expression of CP40 and sequencing of the *cp40* gene

Genomic DNA of *C. pseudotuberculosis* DSM-20689 was extracted using innuPREP Bacteria DNA Kit (Analytikjena Biometra, Göttingen, Germany). The coding sequence of *cp40* was amplified by PCR using the oligonucleotide primers 5′-TGT-AG*C-CAT-GG*G-CGA-GTC-TGC-AAC-CTT-3′ and 5′-GAA-AGG-AAA-ACT-*GGA-TCC*-TCT-AGA-ACC-AGT-TGG-3′ (The restriction sites for NcoI and BamHI are italic). The 1140 bp PCR product was digested with the restriction enzymes NcoI and BamHI (Thermo Fisher Scientific, New York, NY), and ligated into pMAL-c5X-His vector (New England Biolabs, Berkeley, California) using DNA ligase T4 (Thermo Fisher Scientific). The pMAL-c5X-His vector encodes both Maltose-binding protein (MBP) and a 6xhistidine (His) tags Generated pMAL-c5X-His-cp40 plasmid was transformed to *E. coli* Top10 chemically competent cells. Plasmids with correct insert were transformed into the *E. coli* expression strain BL21 (DE3) pLysE. Expression of MBP-cp40-His was induced by 0.1 mM isopropyl-β-D-1-thiogalactopyranoside (IPTG) (VWR International, Radnor, PA) for 2 h at 37 °C. The protein CP40 was purified from the pellet of harvested bacteria according to manufacturer’s protocol using ProteoSpin™ Inclusion Body Protein Isolation Maxi Kit (Norgen Biotek CORP. Thorold, Canada). Purified CP40 protein was dialyzed against PBS at 4 °C.

The *cp40* gene in DSM-20689 was sequenced by Sanger sequencing of overlapping PCR products using the Lightrun sequencing service at GATC Biotech (Konstanz, Germany).

### *N-*glycan hydrolysis assay

To analyze the activity of CP40 on *N-*glycans 0.5 μg of recombinant CP40 was incubated with 1 μg of human IgG and incubated at 37 °C for 2 h. As a control 0.5 μg of MBP and PNGase F also were incubated with IgG in the same condition. The reaction was analyzed by 4–12 % stain-free SDS-PAGE (Bio-Rad Laboratories, Hercules, CA). Proteins were transferred to a PVDF membrane (Bio-Rad Laboratories). Lectin blot was carried out using a Trans-Blot Turbo Transfer system (Bio-Rad Laboratories). The membranes were blocked in lectin buffer (10 mM Hepes, 0.15 M NaCl, 0.1 % Tween 20, 0.01 mM MnCl_2_ and 0.1 mM CaCl_2_, pH 7.5) for 20 min and incubated with 2 μg ml^−1^ of fluorescein labeled LCA (*Lens culinaris* agglutinin) (Vector Laboratories, Burlingame, CA) in lectin buffer for 45 min. The membranes were washed three times with lectin buffer and visualized using a Chemidoc XRS (Bio-Rad Laboratories). In addition 1 μg ovine, equine, bovine and caprine IgG were incubated with 0.5 μg of recombinant CP40 or EndoS independently at 37 °C for 2 h. In parallel 1 μg of all subclasses of human IgG (Sigma-Aldrich, St. Louis, MO) (Calbiotech, CA, for IgG2) independently were incubated with 0.5 μg CP40 in PBS at 37 °C overnight. The reactions were analyzed by 4–12 % stain-free SDS-PAGE and LCA blot as described above.

### Analysis of glycosidase activity of native CP40 in bacterial culture

*C. pseudotuberculosis* DSM-20689 was grown on blood agar plates at 37 °C. Several colonies were inoculated in 100 ml BHI medium and incubated at 37 °C. After 4 h, 500 μl human plasma was added to the culture medium. Supernatant (1 ml) was collected from the bacterial culture after 8, 10, 12 and 24 h. IgG was purified from the samples using Ab spin trap (GE Healthcare, Little Chalfont, UK) and analyzed by 4–12 % stain-free SDS-PAGE and LCA blot as described above. As a control, 1 μg untreated human IgG was run on the gel. The optical density of the bacterial culture was measured at 600 nm at each time point.

### Chitinase assay and substrate specificity of CP40

The fluorogenic substrate 4-methylumbelliferyl-*N*-acetyl-β-D-glucosaminide (4MU-GlcNAc; 0.2 mM) (Sigma–Aldrich) was incubated with 2 μg of recombinant CP40 in a total volume of 100 μl PBS. As controls, 0.3 mU of a chitinase from *Streptomyces griseus* (Sigma-Aldrich), 2 μg EndoSd or 2 μg MBP were included. All reactions were incubated for 1.5 h in a black 96-well plate (Thermo Fisher Scientific) at 37 °C. Addition of 100 μl of 0.1 M glycine (pH 10) stopped the reactions. The assays were carried out using 4 replicates for each reaction. Absorbance at 355/460 nm was measured using spectrophotometer and data are shown as mean ± SD. Different response in absorbance were analyzed statistically using an One-Way ANOVA followed up by Tukey’s multiple comparisons test, where differences were considered significant if *p* < 0.05.

In order to check the substrate specificity of the enzyme, 0.5 μg of recombinantly expressed CP40 was incubated with 1 μg of different glycoproteins such as IgA (Calbiotech), IgE, IgD, IgM, Fetuin, α-1-acid glycoprotein (AGP), and human lactoferrin (hLF) (all Sigma–Aldrich) for 2 h at 37 °C. All samples were incubated with PNGase F under the same conditions as a control. All reactions were analyzed by stain-free SDS-PAGE (Bio-Rad Laboratories). In parallel, 1 μg of bovine, caprine, and equine IgG (Sigma-Aldrich) were incubated with 0.5 μg CP40 or EndoS overnight at 37 °C. In addition 1 μg of all subclasses of human IgG (Sigma-Aldrich, Calbiotech for IgG2) were independently incubated with 0.5 μg CP40 in PBS at 37 °C overnight. The results were analyzed by SDS-PAGE and LCA blot.

### Sequence analysis and similarity tree

The sequence of CP40 from *C. pseudotuberculosis* DSM-20689 was compared with EndoS and different bacterial endoglycosidases and serine proteases. Similarity tree and CP40 active site comparison were generated using the ClustalW alignment in the MacVector software suite (version 15.0.3 (34)) (MacVector, Apex, NC). A reconstruction of a phylogenetic tree was performed using the neighbor-joining systematic method with uncorrected *p*-values. To validate the tree, bootstrapping with 10,000 replications was used.

### Gelatin zymography

To prepare zymogram, 0.1 % gelatin was embedded in 10 % SDS-polyacrylamide gel. Recombinant CP40 (1 μg) or supernatant of *Pseudomonas aeruginosa* as a control were loaded on gel and run under non-reducing conditions. The zymogram was washed with 2.5 % Triton X-100 (Sigma-Aldrich) for 1 h and incubated in an enzyme assay buffer (50 mM Tris– HCl (pH 7.5), 5 mM CaCl_2_, 200 mM NaCl, 1 μM ZnCl_2_) overnight at 37 °C. The gel was stained in Coomassie brilliant blue and then destained with 30 % methanol and 10 % acetic acid.

### Analysis of released IgG glycans by HPLC

*N*-glycans were released from 50 μg of human and ovine IgG by incubation with 2 μg PNGase F, 2 μg EndoS or 5 μg CP40 overnight at 37 °C. Glycans were fluorescently labeled with 2-AB (2-aminobenzamide) (Sigma-Aldrich) and excess labeling reagents removed by normal phase PhyTips (PhyNexus, San Jose, CA). HILIC separation of 2-AB labeled glycans was performed on an Infinity 1260 HPLC System equipped with an Advanced Bio Glycan Map column (2.1 mm × 150 mm 2.7 μm) and a fluorescent detector (all Agilent Technologies, CA). The column temperature was kept at 60 °C and the flow rate set to 0.5 ml min^−1^ using 100 mM ammonium formate (pH 4.5) against acetonitrile with ammonium formate increasing from 15 to 25 % from 0 to 5 min, then 25–36 % from 5 to 35 min. Fluorescence detection was achieved using excitation and emission wavelengths of 330 nm and 420 nm respectively. The released IgG *N-*glycan chromatograms has been well established and assigned [29]. In order to analyze sequence, composition and linkage specificities of the glycans, exoglycosidase digestion arrays were performed (Prozyme, Hayward, CA). Non-reducing end glycan residues were specifically removed using 1 U ml^−1^ ABS (*Arthrobacter ureafaciens* sialidase) to remove terminal sialic acid. Terminal galactose and GlcNAc were removed using 0.5 U ml^−1^ BTG (bovine testes β-galactosidase) and 4 U ml^−1^ GUH (*Streptococcus pneumoniae* hexosaminidase) respectively. To remove core fucose, 1 U ml^−1^ BKF (Bovine kidney fucosidase) was used.

## Results

### CP40 is similar to EndoS-like enzymes

CP40 has been described as a serine protease, but when we searched the protein databases, the protein did not show any significant sequence similarity with any known proteases except for nearly identical corynebacterial proteins that most likely are annotated based on the original characterization of CP40 (data not shown). Instead, CP40 shows similarity to bacterial endoglycosidases and contains a putative glycoside hydrolase family 18 (GH18) domain. We sequenced the *cp40* gene in the DSM-20689 used in this study, and this revealed that the coding sequence only differs in one nucleotide leading to one amino acid substation (proline instead of histidine at position 105) compared to *cp40* in WA1030 used in the original characterization of the enzyme [27].

**Fig. 1:**
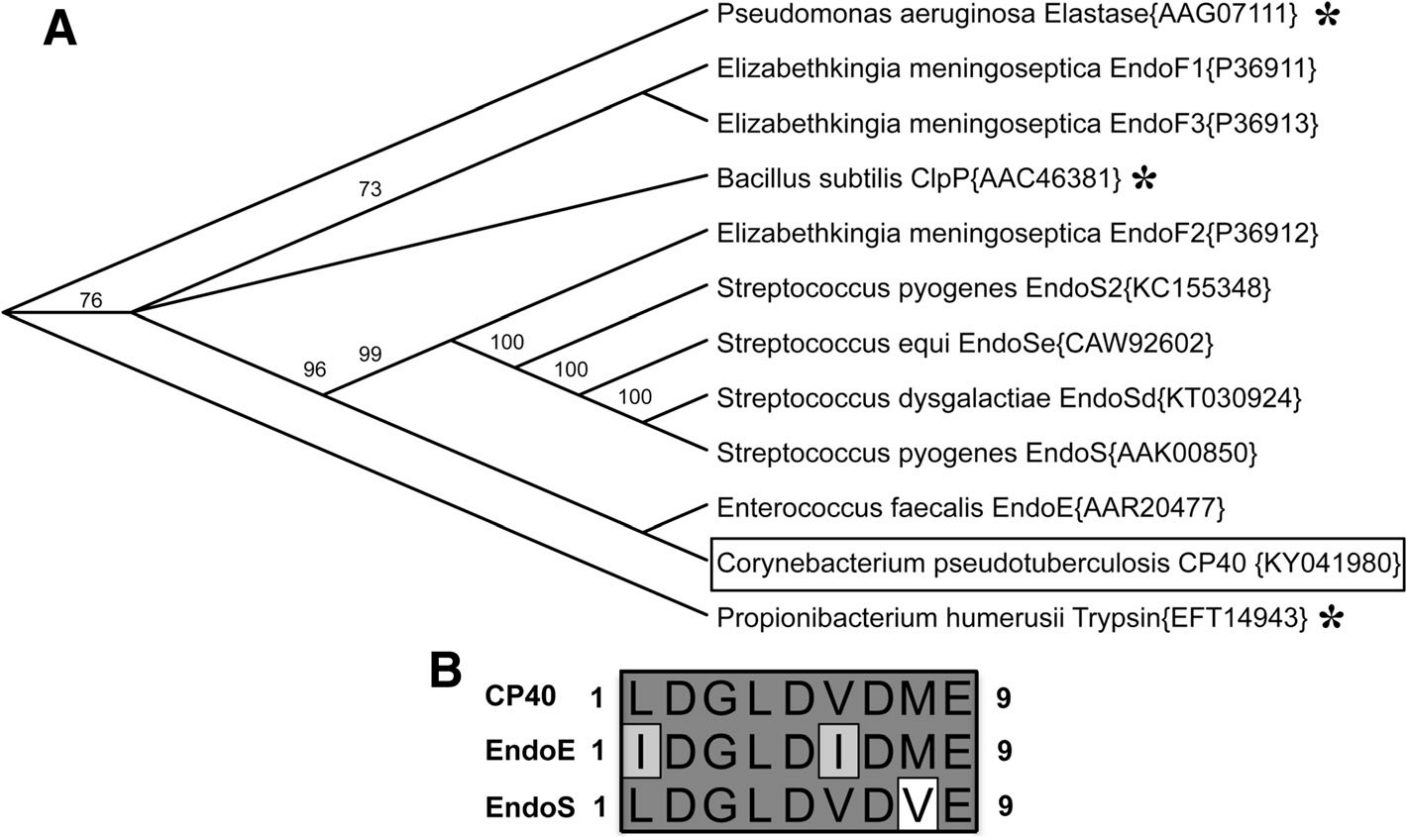
CP40 is similar to bacterial endoglycosidases. **a**. The amino acid sequence of CP40 was aligned with a number of endoglycosidases and serine proteases from different bacterial species. CP40 is enclosed by a rectangle. A phylogenetic tree was reconstructed using the neighbor-joining method, validated by bootstrapping (10,000 replications) generating a consensus tree. Numbers on branching points shows the percentage of resampling trees supporting the consensus tree. GenBank accession numbers of the proteins are indicated in front of the name of each enzyme. The asterisks indicate the serine proteases. **b**. The putative family 18 glycosyl hydrolase active site of CP40 was aligned with the verified active sites of EndoE from *E. faecalis* and EndoS from *S. pyogenes*

We aligned the sequence of CP40 with a number of bacterial endoglycosidases as well as serine protease sequences. The reconstructed phylogentic tree clearly shows that CP40 clusters with the endoglycosidases rather than the proteases (Fig. 1, panel a). Furthermore, CP40 forms a distinct subgroup with the most similar enzyme, EndoE from *Enterococcus faecalis* (Fig. 1, panel a) [30].

GH18 enzymes contain a conserved consensus sequence motif (LIVMFY)-(DN)-G-(LIVMF)-(DN)-(LIVMF)-(DN)-X-E, where the terminal glutamic acid is essential for enzymatic activity [31, 32]. When aligning a putative GH18 active site in CP40 with the most similar enzymes EndoE and EndoS, it becomes clear that CP40 has a perfect GH18 motif that differs only in one or two amino acids as compared to the active site of EndoS and EndoE, respectively (Fig. 1, panel b). The active sites of both EndoS and EndoE have been confirmed by site-directed mutagenesis, and interestingly both enzymes have activity on the *N-*linked glycans in IgG Fc [13, 30]. Taken together this analysis clearly indicates that CP40 could be a GH18 enzyme and we addressed this hypothesis experimentally by testing for general chitinase activity, or more specific glycan hydrolyzing activity on host glycoproteins.

### CP40 and *C. pseudotuberculosis* have glycosidase activity on IgG

**Fig. 2:**
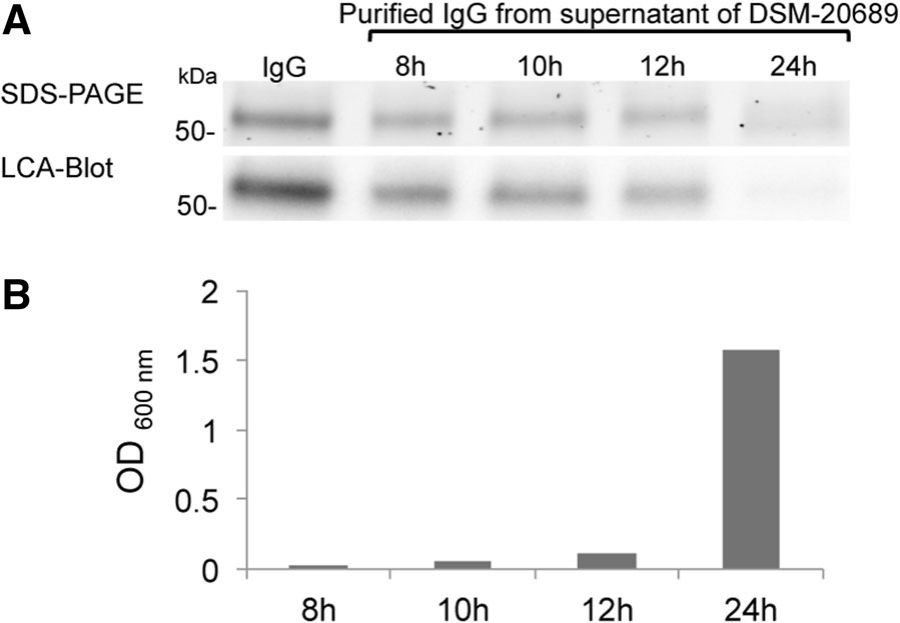
IgG *N-*glycan hydrolyzing activity can be detected during growth of *C. pseudotuberculosis*. **a**. *C. pseudotuberculosis* was grown in 100 ml BHI. After 4 h, 500 μl of human plasma was added to the culture. 1 ml samples were taken from the bacterial culture after 8, 10,12, 24 h. IgG was purified from the culture and IgG glycan hydrolysis was analyzed by SDS-PAGE and LCA blot. **b**. The OD of bacterial culture was measured at 600 nm at the indicated time point

**Fig. 3:**
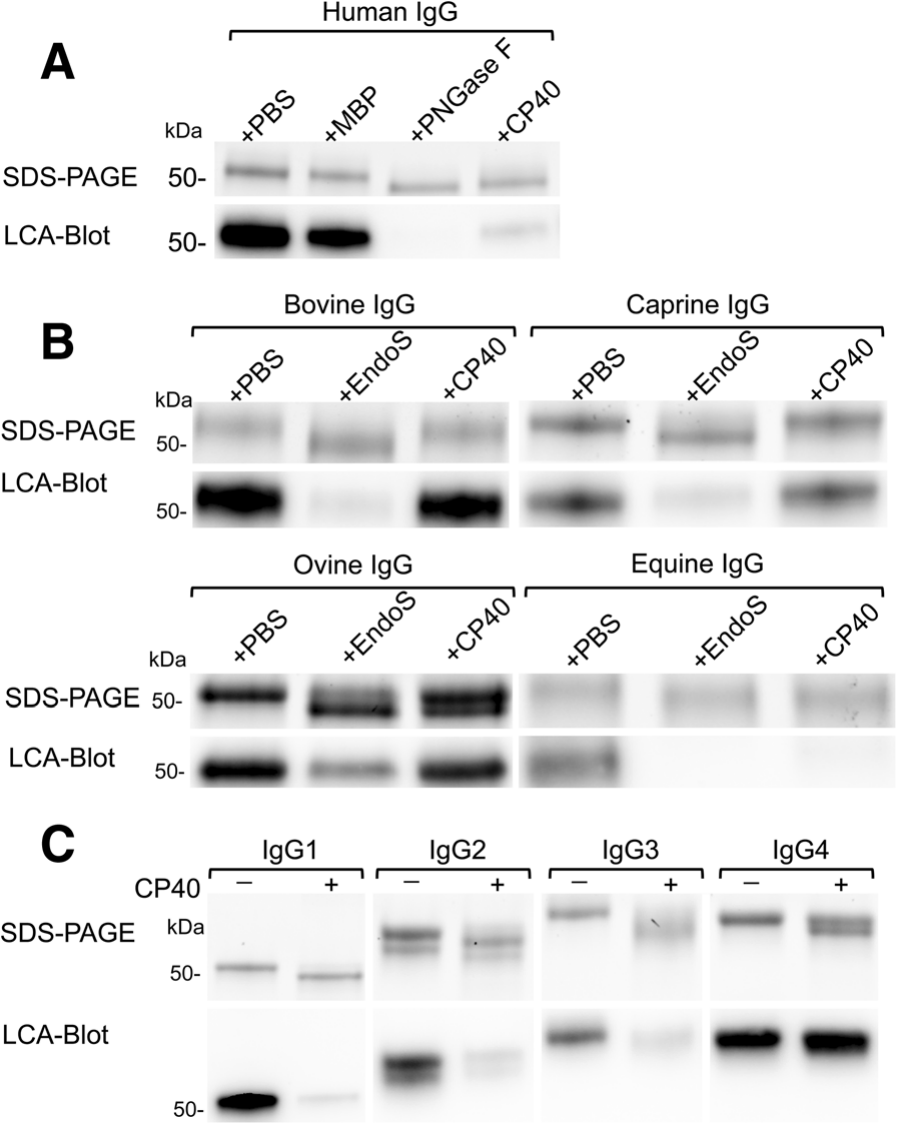
CP40 hydrolyzes N*-*linked glycans on human and animal IgG. **a**. Recombinant CP40 (0.5 μg) was incubated with to 1 μg of human. As controls, 0.5 μg of MBP and PNGases F were incubated with human IgG. **b**. ovine, equine, bovine and caprine IgG (1 μg) were incubated with 0.5 μg of CP40 and EndoS. **c**. All subclasses of human IgG (1 μg) were incubated with 0.5 μg of recombinant CP40. All of the reactions were at 37 °C for 2 h and IgG glycan hydrolyzing was analyzed by SDS-PAGE and LCA blot

Based on the clear relationship between CP40 and the known IgG glycan hydrolases EndoS and EndoE, we speculated that IgG could be a substrate also for CP40. As CP40 is secreted [26], we cultured *C. pseudotuberculosis* DSM-20689 in medium supplemented with 0.5 % human plasma. After 8,10,12 and 24 h of culture, IgG was purified from the culture supernatant and analyzed by SDS-PAGE and LCA blot (Fig. 2, panels a and b). After 24 h when the bacteria were in late exponential phase, a clear mass shift of IgG and a lack of LCA signal could be observed. However, the LCA signals seemed to gradually diminish from the 10 h sample to be completely gone in the 24 h sample. This indicates that *C. pseudotuberculosis* does secrete a glycoside hydrolase. To test if this activity is indeed mediated by CP40, we recombinantly expressed the enzyme as a maltose-binding protein (MBP)-fusion protein in *E. coli* and incubated the enzyme with pooled polyclonal human IgG. Glycan hydrolyzing activity was subsequently analyzed by SDS-PAGE and LCA blot. MBP alone, and PNGase F were used as controls. A mobility shift of the IgG heavy chain on SDS-PAGE and a corresponding lack of signal on the LCA blot (LCA recognizes α-linked mannose) were observed when IgG was incubated with CP40 and PNGase F, but not for MBP and the buffer control (Fig. 3, panel a).

Since *C. pseudotuberculosis* is primarily an animal pathogen, we investigated whether animal IgGs are substrates for CP40. While EndoS was able to hydrolyze the glycan chain on all tested animal IgGs, as judged by a ~3 kDa shift of the heavy chain in combination with a loss of LCA signal, CP40 was only able to fully hydrolyze equine IgG and partially hydrolyze ovine IgG, but showed no activity on bovine or caprine IgG (Fig. 3, panel b). To further evaluate the glycan hydrolyzing activity of CP40, we incubated CP40 with the four subclasses of human IgG. This revealed that CP40 has activity versus all human IgG subclasses, albeit the hydrolysis of IgG4 was incomplete (Fig. 3, panel c).

### CP40 is not a general chitinase or broad spectrum endoglycosidase

**Fig. 4:**
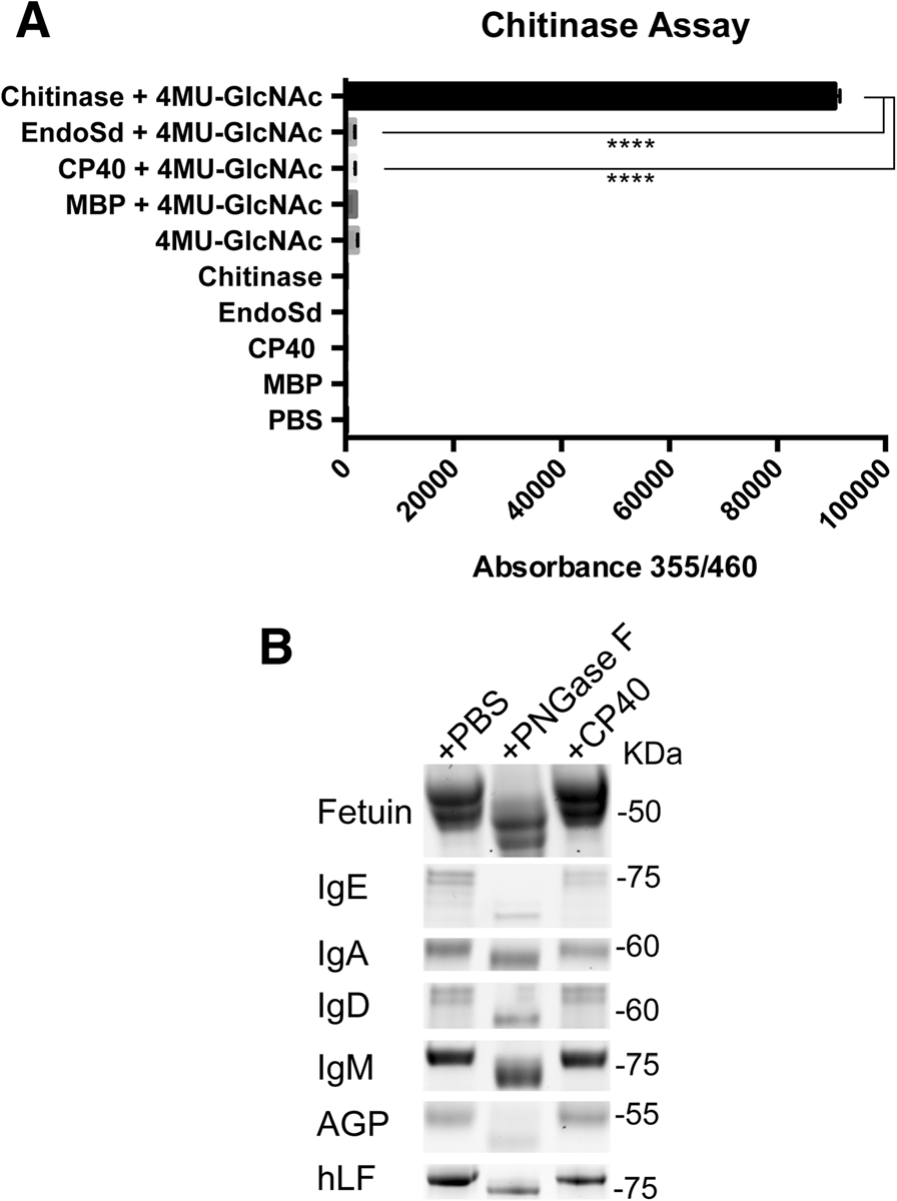
CP40 is not a general chitinase and does not show glycosidase activity on several glycoproteins. **a**. CP40, EndoSd, MBP and a chitinase from *S. griseus* were incubated with the fluorescent substrate 4MU-GlcNAc for 1 h and fluorescence was measured at 355/460 nm. The experiments were performed in five replicates. Data are presented as mean ± S.D. The absorbance was analyzed statistically by One-way Anova followed up by Tukey’s multiple comparisons test. ****P < 0.0001. **b**. Recombinant CP40 (0.5 μg) was incubated with fetuin, IgE, IgA, IgD, IgM, AGP, hLF for 2 h at 37 °C and glycan hydrolyzing activity were analyzed by SDS-PAGE

In order to investigate if the observed activity of CP40 on IgG could be attributed to a general endochitinase activity, CP40 was incubated with the well-characterized fluorogenic chitinase substrate 4 MB-GlcNAc. We have recently established that the related enzyme EndoS and its homolog EndoSd are not general endochitinases [33]. Therefore EndoSd was used as a negative control, while a verified chitinase from *Streptomyces griseus* was used as a positive control. This revealed that there is no detectable activity of CP40 on 4 MB-GlcNAc, clearly indicating that CP40 is not a general endochitinase (Fig. 4, panel a).

To elucidate if CP40 has a broader activity on *N-*linked glycans on glycoproteins, the enzyme was tested for activity against the purified glycoproteins IgA, IgE, IgD, IgM, fetuin, α_1_-acid glycoprotein (AGP), and human lactoferrin (hLF). As a positive control PNGase F, known to hydrolyze all mammalian *N-*linked glycans, was used. After incubation, samples were analyzed SDS-PAGE to assess possible size shifts. This revealed that no obvious size shifts could be observed when glycoproteins were incubated with CP40, in sharp contrast to PNGase F, where clear size shifts could be observed (Fig. 4, panel b). These results indicate that CP40 has no major activity on the glycoproteins tested here.

### CP40 is not a protease

**Fig. 5:**
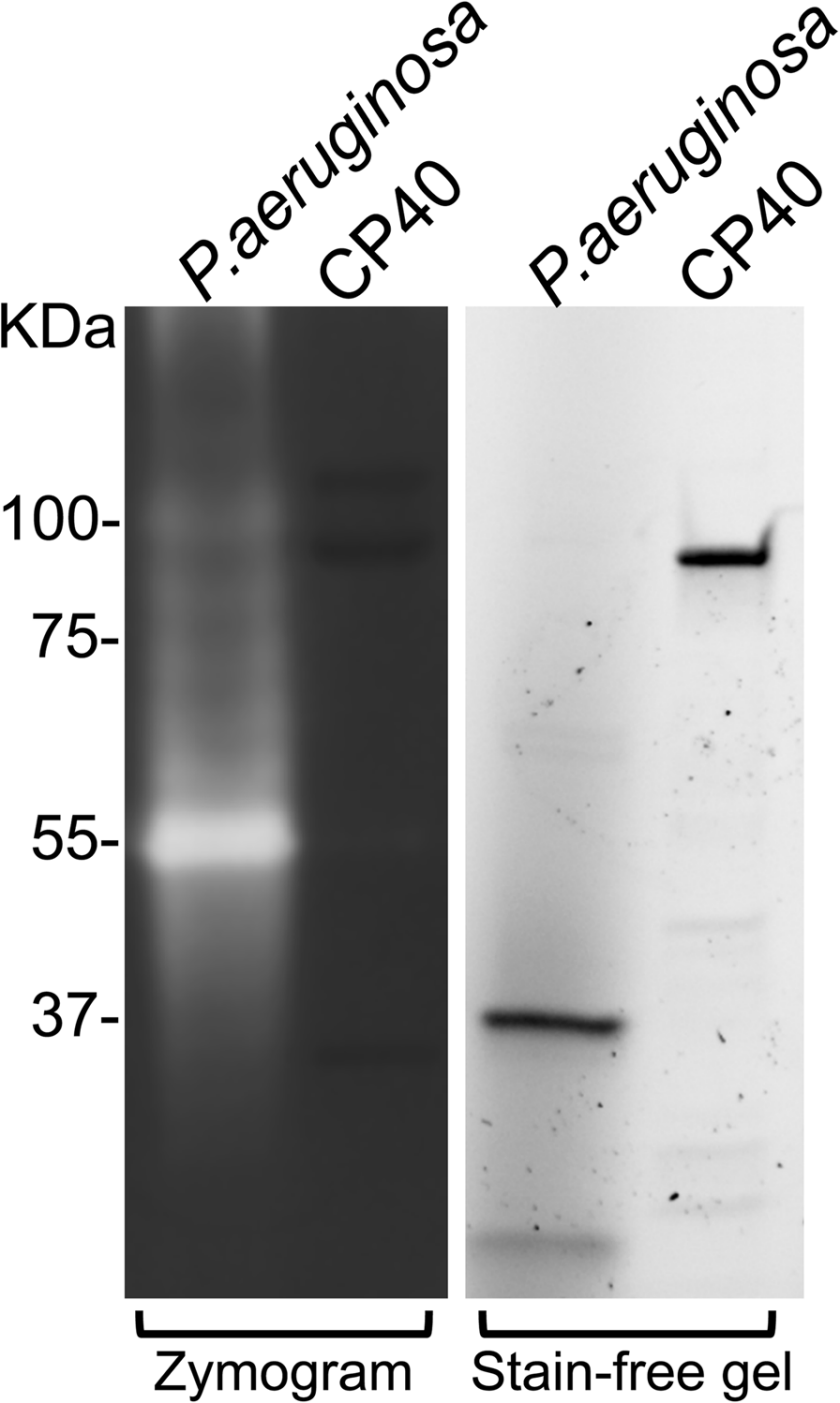
CP40 is not a protease. Recombinant CP40 (1 μg) was loaded on a zymogram (0.1 % gelatin SDS-PAGE) and stain free gel in parallel. Supernatant of *P. aeruginosa* was used as a positive control

During the initial characterization of CP40, a recombinant protein with a thrombin cleavable tag was used. Based on gelatin zymography of the thrombin cleaved recombinantly expressed CP40 and culture supernatants from *C. pseudotuberculosis* strain WA1030 and the use of specific protease inhibitors, it was concluded that CP40 is a serine protease [27]. We attempted to repeat this experiment using recombinantly expressed CP40 and essentially the same gelatin zymography method. This revealed that there was no detectable clearing zone around CP40 in the zymogram, while there was a distinct clearing zone in the positive control corresponding to an elastase in *Pseudomonas aeruginosa* culture supernatant (Fig. 5).

### CP40 hydrolyzes biantennary, but not bisecting glycans on IgG

**Fig. 6:**
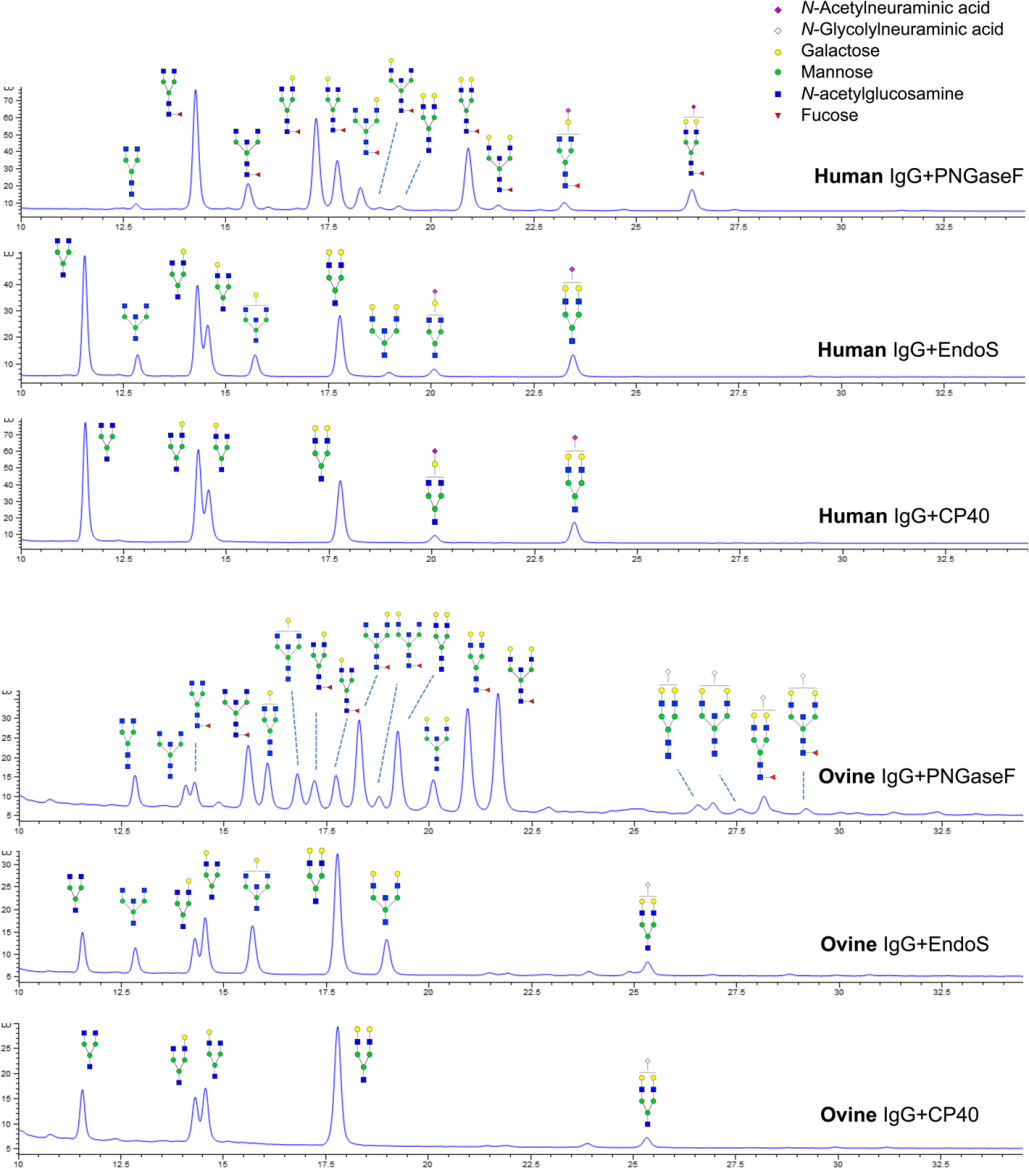
CP40 hydrolyzes biantennary, but not bisecting glycans on IgG glycans from human and ovine IgGs. Glycans were released from human and ovine IgG by PNGase F, EndoS and CP40 respectively, glycans were with 2AB and analyzed by HILIC. The structures annotated according to the digested glycan using a series of defined exoglycosidase. The released biantennary glycans from IgG and the cleavage site of the glycan chain are shown. Monosaccharide identities are shown to the right in the middle and conform to the standard colors and shapes as devised by the Consortium for Functional Glycomic (www.functionalglycomics.org)

In order to elucidate the glycoform preferences of CP40 in more detail, we turned to HPLC analysis of released glycans. CP40, EndoS, or PNGase, were used to release glycans from human and ovine IgGs (Since *C. pseudotuberculosis* causes CLA mostly in sheep), and glycans were subsequently fluorescently labeled and analyzed using a glycan dedicated hydrophilic interaction liquid chromatography (HILIC) system. Comparison of the resulting chromatograms from CP40 and EndoS indicate that the glycan cleavage site of CP40 is identical to that of EndoS (Fig. 6). Furthermore, the chromatograms of CP40 generated glycans indicate that this enzyme can hydrolyze all biantennary glycoforms from human and ovine IgGs with the notable exception of structures containing a bisecting GlcNAc (Fig. 6).

## Discussion

CLA in sheep caused by *C. pseudotuberculosis* is a globally important disease that has proven to be difficult to control [34]. Control of the disease is of substantial commercial interest since CLA causes economical losses due to reduced productivity in the dairy, meat and leather industries [24]. Since the vaccines against the different virulence factors of the bacteria such as phospholipase D (PLD) toxin do not induce strong immunity to CLA, researchers are looking for new protective and immunogenic antigens [35]. There are also difficulties associated with using current commercial vaccines due to their limited efficacy as well as regulations in some countries [35]. Towards that end, CP40 is highly interesting since it has been established as a major antigen expressed during infection and animals seroconvert to this protein. Most importantly, CP40 immunization generates protective immunity in both sheep and mice [26, 36, 37]. CP40 has undoubtedly been established in the literature as one the most important secreted proteins from *C. pseudotuberculosis*. Even though is it not essential for vaccine strategies to completely understand the function of the antigen, molecular characterization can strengthen the basis for targeting the antigen, and most importantly, can increase the understanding of the basic biology of the infective agent.

We have during a number of years been specializing in the characterization of bacterial endoglycosidases with activity of host glycoproteins. During that CP40 has again and again showed up as an odd bird when doing searches with one of our characterized endoglycosidases (data not shown). Furthermore, recent whole genome sequencing projects of different corynebacteria have started to question the annotation of CP40 as a protease and instead link it to endoglycosidase activity [28, 38]. This in combination with our bioinformatic analysis of CP40 strongly suggested that this protein is not a protease, but an endoglycosidase belonging to the GH18 family. Since the protein is fairly small, the likelihood of an additional protease domain is very low. However, since annotation of function based on bioinformatics can be quite ambiguous, clear experimental evidence was needed in order to annotate a completely new function to CP40. When attempting to reproduce the original finding that CP40 has activity against gelatin in zymography [27], we were not able to see any protease activity of the protein (Fig. 5). Size shift of IgG on SDS-PAGE and lack of signal on LCA blot during bacterial growth, suggests that *C. pseudotuberculosis* express IgG glycan hydrolyzing activity (Fig. 2). This could be attributed to expression of CP40, but we cannot formally exclude that the bacteria express additional enzymes with activity on IgG. Our interpretation is that CP40 is not a protease, and that the previously detected serine protease activity is most likely due to remaining thrombin in the recombinant CP40 preparation, and an unrelated serine protease activity in the culture medium of the *C. pseudotuberculosis*.

Bacterial endoglycosidases has been extensively used as tools in glycobiology research, but have been somewhat overlooked in pathogenesis research [10]. However, since glycobiology of the immune system, or glycoimmunology, is getting more attention, bacterial modification of glycoproteins beyond mere nutrient acquisition is indeed relevant to study in detail [9]. The activity of GH18 enzymes ranges from chitinase activity that hydrolyzes basically any polymer of GlcNAc, to more specific enzymes that hydrolyses the chitobiose core in certain glycoforms on any glycoprotein, and enzymes that are both glycoform and protein specific as exemplified by EndoS, EndoS2, EndoE, and EndoSd [12, 30, 33, 39]. Based on the similarities to the verified IgG glycan hydrolases EndoE and EndoS, we first analyzed if CP40 could hydrolyze glycans in IgG from different sources, and in the case of human IgG, also of different subclasses (Fig. 3). This revealed that bovine and caprine IgG treated with CP40 were intact, while equine IgG was complete hydrolyzed. All animal IgGs used in this study contain *N*-glycosylneuraminic acid (NGNA) at the termini of the IgG glycan chain [40]. Furthermore, the total content of carbohydrate in caprine IgG is much lower than human and ovine IgG. The amount of core fucosylated, terminally galactosylated and bisecting oligosaccharides on caprine IgG is also very low compared to human and ovine IgG [40]. Equine IgG has no detectable bisecting GlcNAc on the *N-*glycan chain and bovine IgG has a very low amount of bisecting glycans on IgG [40].

As mentioned above, many GH18 enzymes have activity on chitin alone, or in the chitobiose core of *N-*linked glycans, irrespective of the protein backbone. We therefore tested if CP40 had activity on the widely used endochitinase substrate 4MU-GlcNAc and compared to verified chitinases. The results clearly showed that CP40 is not a general chitinase (Fig. 4, panel a).

CP40 seemed to have some preference for IgG, and there were no detectable activity on a number of other glycoproteins harboring *N-*linked glycan, including some of the other immunoglobulin isotypes (IgA, IgD, and IgA). However, GH18 enzymes can also display preferences for certain glycoforms, as exemplified by EndoF_1–3_ and EndoS/EndoS2 [41, 42]. We therefore turned to HPLC analysis of released glycans that has the potential elucidate the glycoform specificity in more detail. HPLC chromatograms of CP40 released glycans from human and ovine IgGs showed that this enzyme could only hydrolyze biantennary structures and not bisecting GlcNAc (Fig. 6). This glycoform selectivity closely resembles that of the EndoS homolog EndoSd from *S. dysgalactiae* [33]. Ovine IgG contains bisecting GlcNAc and high mannose structures compared to human IgG; it also has terminal NGNA instead of *N-*acetylneuraminic acid (NANA) that is found in human IgG [40]. Previous studies have suggested that carbohydrate binding domains are important for releasing bisecting structures by EndoS, since EndoS lacking carbohydrate binding domain releases bisecting GlcNAc slower than other glycoforms [43]. Since CP40 is a much smaller enzyme than EndoS and lacking putative carbohydrate binding domains, this could potentially explain the lack of activity against IgG glycans with bisecting GlcNAc.

## Conclusions

We believe that we have presented very strong evidence for that CP40 is a not a serine protease, but rather an endoglycosidase with activity on immunologically important host proteins. Furthermore, during the final stages of preparation of this manuscript, a review about virulence factors of *C. ulcerans* identified the CP40 homolog in this species as a GH18 endoglycosidase with similarity to the α domain of EndoE from *E. faecalis* [44]. Similar endoglycosidases in other bacterial pathogens have been suggested to be involved in evasion of the adaptive immune response, but we have not direct evidence for that CP40 hydrolyzes IgG glycans in vivo and contributes to immune evasion. However, a solid body of work done on CP40 by other research groups shows that CP40 is indeed expressed in vivo*,* and that immunity towards this protein provides protection against infection. This makes our molecular characterization of this protein relevant, and may aid in the continued development of vaccines and antimicrobial strategies to combat infections with *C. pseudotuberculosis.*

## Abbreviations

2-AB: 2-aminobenzamide
4MU-GlcNAc: 4-methylumbelliferyl-*N*-acetyl-β-D-glucosaminide
ABS: *Arthrobacter ureafaciens* sialidase
AGP: α-1-acid glycoprotein
BKF: Bovine kidney fucosidase
BTG: Bovine testes β-galactosidase
*C. pseudotuberculosis*: *Corynebacterium pseudotuberculosis*
*E. coli*: *Escherichia coli*
*E. faecalis*: *Enterococcus faecalis*
FcγR: IgG-Fc receptor
GH18: glycoside hydrolase family 18
GlcNAc: *N*-acetylglucosamine
GUH: *Streptococcus pneumoniae* hexosaminidase
HILIC: Hydrophilic interaction liquid chromatography
hLF: Human lactoferrin
Ig: Immunoglobulin
IPTG: Isopropyl-β-D-1-thiogalactopyranoside
LB: Luria broth
LCA: *Lens culinaris* agglutinin
MBP: Maltose-binding protein
NANA: *N-*acetylneuraminic acid
NGNA: *N*-glycosylneuraminic acid
PLD: phospholipase D
